# An Integrative Analysis of microRNA and mRNA Expression—A Case Study

**DOI:** 10.4137/cin.s633

**Published:** 2008-06-17

**Authors:** Li-Xuan Qin

**Affiliations:** Department of Epidemiology and Biostatistics, Memorial Sloan-Kettering Cancer Center, New York, New York, U.S.A

**Keywords:** clustering, expression, microarray, microRNA

## Abstract

**Background:**

MicroRNAs are believed to play an important role in gene expression regulation. They have been shown to be involved in cell cycle regulation and cancer. MicroRNA expression profiling became available owing to recent technology advancement. In some studies, both microRNA expression and mRNA expression are measured, which allows an integrated analysis of microRNA and mRNA expression.

**Results:**

We demonstrated three aspects of an integrated analysis of microRNA and mRNA expression, through a case study of human cancer data. We showed that (1) microRNA expression efficiently sorts tumors from normal tissues regardless of tumor type, while gene expression does not; (2) many microRNAs are down-regulated in tumors and these microRNAs can be clustered in two ways: microRNAs similarly affected by cancer and microRNAs similarly interacting with genes; (3) taking let-7f as an example, targets genes can be identified and they can be clustered based on their relationship with let-7f expression.

**Discussion:**

Our findings in this paper were made using novel applications of existing statistical methods: hierarchical clustering was applied with a new distance measure—the co-clustering frequency—to identify sample clusters that are stable; microRNA-gene correlation profiles were subject to hierarchical clustering to identify microRNAs that similarly interact with genes and hence are likely functionally related; the clustering of regression models method was applied to identify microRNAs similarly related to cancer while adjusting for tissue type and genes similarly related to microRNA while adjusting for disease status. These analytic methods are applicable to interrogate multiple types of -omics data in general.

## Background

MicroRNAs (miRNAs) are a class of small non-coding RNAs that are believed to regulate gene expression [1, 2]. The first two miRNAs, lin-4 and let-7, were experimentally discovered in 1993 and 2000 [3, 4]. Since then more than 4300 miRNAs have been identified in plants, animals, and viruses using cDNA sequencing and computational predictions [5–8]. MiRNAs regulate their target genes, through base-pairing, by inducing mRNA degradation and translational repression [1, 2]. In humans, miRNAs might regulate as many as a third of the protein coding genes [9]. MiRNAs are likely to have an important impact on development in various cellular processes, such as cancer. MiRNAs have been shown to be linked to a number of cancer types in several studies on individual miRNAs [10, 11].

Cancer is a complex and heterogeneous disease whose initiation and progression are influenced by a variety of molecular changes [12]. A complete characterization of the genomic changes may help predict the pathologic behavior of cancer. Genome-wide profiling of gene expression has been increasingly applied in clinical settings to understand genomic changes at the mRNA level. Examples can be found in breast, colon, ovarian, and prostate cancer [13–16]. Such knowledge has improved our understanding of cancer biology and facilitated the discovery of new cancer subtypes and new biomarkers for cancer diagnosis, prognosis, and treatment [17, 18].

Large-scale expression profiling has recently become available for miRNAs as well [19]. Profiling methods for miRNA expression are mostly based on glass-slide microarrays [20–22] and the latest development is the bead-based flow cytometry technique [23]. Genome-wide miRNA studies allow the investigation of genomic changes in cancer at the miRNA level and are likely to provide additional clues to the mechanisms of tumorigenesis [23–25]. In particular, when miRNA and mRNA expression are both measured on the same samples, an integrative analysis can be performed to compare miRNAs and mRNAs profiles and to study their interaction patterns. The goal of this paper is to demonstrate three aspects of such an integrative analysis through novel applications of existing statistical methods.

In this paper, we will focus on the following three aspects of an integrative analysis of mRNA and miRNA expression data, while taking into account of sample phenotype data (for example, tumor *versus* normal samples).

1. Stable sample clustering based on miRNA expression in comparison with that based on gene expression.
2. Identification of cancer-related miRNAs and clustering of these miRNAs into groups that similarly interact with genes and into groups that are similarly affected by cancer.
3. Identification of candidate target genes for a given miRNA and clustering of these genes based on their relationship with miRNA expression and disease status.

We will demonstrate these three aspects of an integrative analysis using a published study of miRNA and mRNA expression in various types of tumor samples [23]. A set of 46 samples, whose miRNA expression and gene expression were both measured, was used in our analysis (Supplementary Table 1). These 46 samples consist of 28 tumor samples belonging to five tissue types and their 18 normal counterparts (>1 normal per tissue type). MiRNAs and genes with truncated values in >10% samples are excluded, which results in 128 miRNAs and 7149 genes in our analysis.

## Results

### Clustering samples

Pioneered by Eisen et al. [26], hierarchical clustering is the most commonly used method for sample clustering using expression profiles. With hierarchical clustering, a distance measure is calculated between the expression profiles of each gene (or gene cluster) pair, and a recursive bottom-up or top-down algorithm is then employed to merge or split genes based on their distance. Examples of distance measures include the Euclidean distance and one minus the Pearson correlation coefficient. Hierarchical clustering does not require the number of clusters to be pre-specified and has nice visualization properties (dendrogram and heatmap). Similar to many other clustering algorithms, a well-recognized drawback of hierarchical clustering, however, is that it always generates a clustering even when there is no real underlying clustering in the data. It is not apparent whether the clustering structure reflects a ‘true’ pattern in the data or is just an artefact of the clustering algorithm. Methods based on resampling have been proposed to evaluate the significance of a clustering [27–29]. These methods simulate perturbations of the original data and assess the stability of the clustering results.

Also based on resampling, Monti et al. proposed a method, called ‘consensus clustering’, that makes use of the resampling results to guide clustering [30]. Briefly, consensus clustering quantifies the agreement among clustering runs over the perturbed data sets, measured by a consensus matrix whose elements are the frequency that two samples are clustered together, and then performs hierarchical clustering using the consensus matrix as similarity matrix.

In the consensus clustering, the co-clustering frequency measure counts co-clustering frequency of two samples among perturbed data sets that include both samples. Instead, we apply the clustering of each perturbed data set to classify samples in the original data set using the nearest-centroid method and then count the frequency of two samples being classified together among all perturbations. We will call this method as ‘stable hierarchical clustering’. We used a partitional clustering method, PAM (partitioning around medoids) [31], to cluster each perturbed data set in this paper. Details of the stable hierarchical clustering method are provided in Method section.

**Figure 1:**
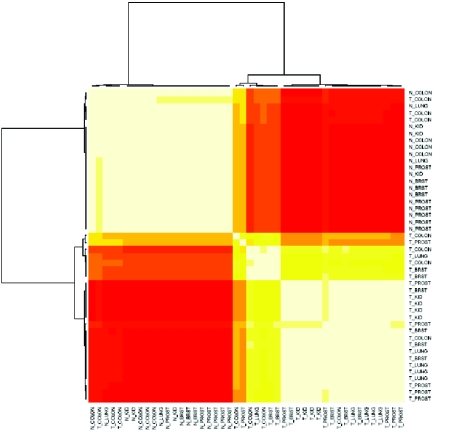

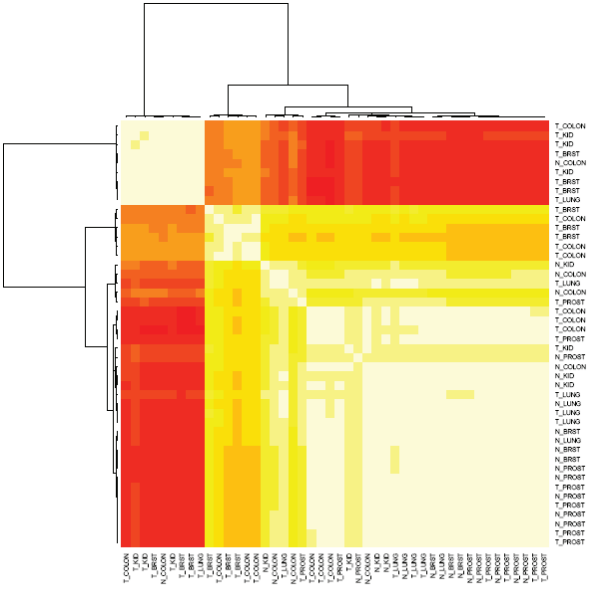
Figure 1a. Sample clustering based on miRNA expression. The heatmap represents the co-clustering frequency between samples: the co-clustering frequency on the diagonal is always 1, as a sample is always clustered with itself. The dendrogram represents the sample clustering using the hierarchical clustering method with the co-clustering frequency as the similarity measure. Each sample is denoted with its disease status (T = Tumor, N = Normal) and tissue type (BRST = Breast, COLON, KID = Kidney, LUNG, PROST = Prostate). Figure 1b. Sample clustering based on gene expression.

We first applied stable hierarchical clustering to identify stable sample clusters based on miRNA expression (Fig. 1a). Interestingly, except for three colon tumors, tumor samples were well separated from normal samples, regardless of tissue type. A potential explanation of the mis-clustering of the three colon tumors is normal tissue contamination, which colorectal cancer is prone to. The three colon tumors were excluded from our subsequent analysis. Nonetheless, this clustering result suggests that miRNA expression has the potential of distinguishing tumors from normal samples for clinical diagnosis.

We also applied stable hierarchical clustering to cluster samples based on gene expression. It did not separate tumors from normal samples as efficiently and it tended to recognize tissue types rather than disease status (Fig. 1b). A possible explanation is the following: (i) only a small number of genes have signals differentiating cancer from normal and a large number of genes are only adding noise, which might obscure or distort the signals; and (ii) miRNAs are upstream in the regulatory network and thus might contain more accurate information about the state of the sample.

Hierarchical clustering has been applied to cluster samples in the original publication by Lu et al. [23]; however, they clustered tumor samples only and discovered clusters reflecting various tumor characteristics. We showed, through subjecting both tumors and normals to clustering and adopting a new distance measure for clustering, that miRNA expression can clearly distinguish tumors from normals, regardless of tissue type. Lu et al. showed the inferiority of mRNA expression in distinguishing GI *versus* Non-GI tumors, while we showed its inferiority in distinguishing tumors *versus* normals.

### Identifying and clustering cancer-related miRNAs

**Figure 2:**
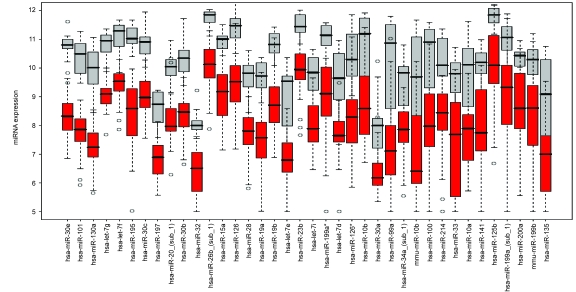
Boxplot for the 38 cancer-related miRNAs among normal samples (grey) and tumor samples (red).

Filtering has been commonly adopted as a useful pre-processing step, to remove uninformative genes and to reduce computational burden, for gene clustering. We applied similar filtering step for miRNA clustering. Specifically, we selected a subset of miRNAs that are related to cancer, by modelling miRNA expression using a per-gene linear regression model with disease status and tissue type as covariates [32] and evaluating the significance of the correlation with disease status using an Empirical Bayesian t test [33]. Among the 128 miRNAs, 89 (70%) were found to be related to cancer at the significance level of 0.001. The 89 miRNAs were all down-regulated in tumors. In the subsequent cluster analysis, we focused on a subset of 38 miRNAs, which had both a small p-value (<0.001) and a large fold change (>3) (Table 1, Fig. 2).

To better understand the grouping structure among these 38 cancer-related miRNAs, we clustered them in two ways. One is to identify groups of miRNAs that similarly interact with genes. The other is to identify groups of miRNAs that are similarly affected by disease status.

**Figure 3:**
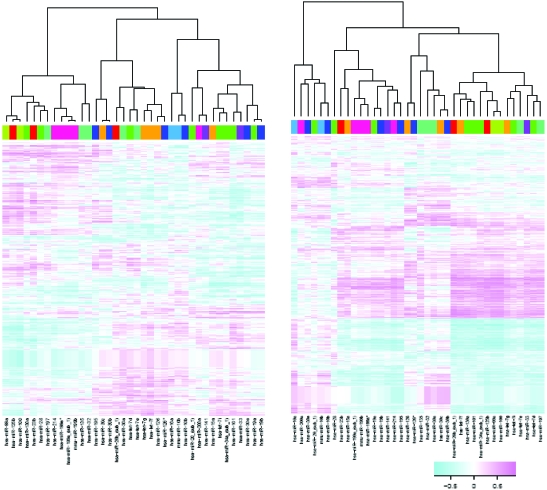
Figure 3a. (left panel)—Hierarchical clustering of the 38 cancer-related miRNAs based on the miRNA-gene correlations among tumor samples. Heatmap represents the miRNA-genes correlations, with columns for miRNAs and rows for genes. Figure 3b. (right panel)—Hierarchical clustering of the 38 cancer-related miRNAs based on the miRNA-gene correlations among normal samples.

MiRNAs were clustered based on their correlation patterns with gene expression. The correlation matrix between miRNA expression and gene expression were calculated and graphically displayed with a heatmap, where rows are genes and columns are miRNAs. MiRNAs were then clustered based on this correlation matrix, so that miRNAs with similar correlation profiles (ie. similar columns of the correlation matrix) were clustered together. For the clustering step, the hierarchical clustering method was employed, with the Euclidean distance as the distance measure. MiRNAs closer on the dendrogram share similar correlation pattern with genes and are thus likely functionally related. The miRNA-gene correlation heatmap and the corresponding miRNA clustering were generated separately for tumor samples and normal samples (Figs. 3a–b). Of note, several let-7 family miRNAs, although belonging to different clusters based on mean expression levels, share similar correlation patterns with gene expression profiles in both tumor and normal tissues.

**Figure 4:**
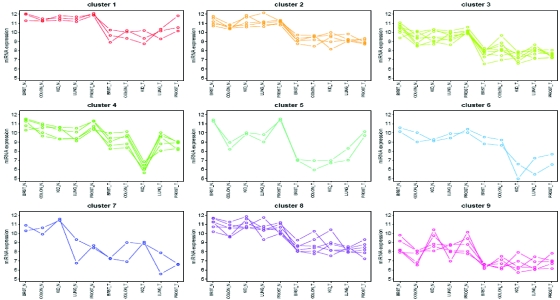
Profile plot for CORM clusters of the 38 cancer-related miRNAs Each panel is for a miRNA cluster and each line is for a miRNA. X axis represents combinations of disease status (T = Tumor, N = Normal) and tissue type (BRST=Breast, COLON, KID = Kidney, LUNG, PROST = Prostate). Y axis is the mean miRNA expression level for a given tissue type and disease status.

MiRNAs were also clustered based on their relationship to disease status. A new clustering method, the clustering of regression models (CORM) method, models expression using regression and assumes that miRNAs in the same cluster share the same regression coefficients [34]. This method tends to provide more stable clustering than K-means clustering, as it explicitly models different sources of variations and bases clustering solely on the systematic variation [35]. Using the CORM method with disease status and tissue type as the covariates, miRNAs were clustered so that miRNAs in the same cluster have similar mean expression among tumor samples and normal samples for each tissue type (Table 1, Fig. 4). It is reassuring to see that variants of the same miRNA tend to belong to the same cluster. For example, let-7f and let-7g are both in cluster 2. Of note, miRNAs in cluster 2 (let-7f, let-7g, miR-15a, miR-30c, and miR-126) are expressed at a similar level among normal samples and down-regulated at a similar level among tumors, regardless of tissue type. In addition, miRNAs in cluster 4 (miR-199a, miR-199b, miR-200a, and miR-214) are significantly down-regulated in kidney tumors. Interestingly, miR-200a was first cloned in mouse kidney tissue and its expression was confirmed in humans [36].

### Identifying and clustering target genes for let-7f

MiRNAs are thought to negatively regulate mRNA in one of two ways depending on the degree of complementarity between the miRNA and its target [37]: (1) miRNAs that bind perfectly to their target’s coding sequence are thought to result in mRNA degradation, and (2) miRNAs that bind with imperfect complementarity to the 3′ UTR block target gene expression at the level of protein translation.

Target gene prediction is an important but complicated task for miRNA studies [38]. Several algorithms have been proposed for miRNA target prediction, which mostly rely on the assumption of base pairing and evolutionary conservation. Examples of the sequence-based prediction algorithms include MiRanda, PicTar, and TargetScanS [39–42]. A large number of miRNA targets are still unknown [43]. MiRNA expression profiling provides an alternative for identifying target genes, especially those targeted through degradation, by correlating miRNA and gene expression. It can potentially provide *in vivo* evidence of gene targeting, as opposed to the *in silico* evidence provided by the sequence-based prediction algorithms.

**Figure 5:**
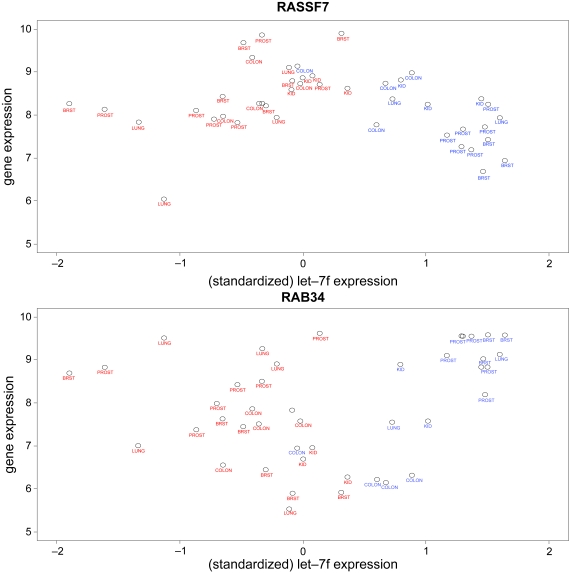
Figure 5a. Scatter plot for RASSF7 expression *versus* let-7f expression. X axis is let-7f expression and Y axis is RASSF7 expression. Tissue type is labeled under each point. Color of the label represents disease status: red for tumor and blue for normal. Figure 5b. Scatter plot for RAB34 expression *versus* let-7f expression.

We took let-7f as an example to demonstrate how to identify and cluster miRNA target genes using miRNA and gene expression. For each gene, the expression level is modeled using a linear regression model with let-7f expression, disease status, and their interaction as the covariates. This full model is then compared to a reduced linear regression model with disease status as the covariate, using a likelihood ratio test, to evaluate the association between gene expression and let-7f expression. A set of 178 genes showed significant association with let-7f expression using a significance cut-off of p-value = 0.001 (Supplementary Table 2). These 178 are potential let-7f target genes, with a false discovery rate of about 0.015 [44]. These 178 genes are enriched in nucleic acid binding and regulation of DNA replication or transcription; a number of the predicted target genes are related to cancer, such as RASSF7, RAB34, ARAF, BCL2L14, MLL3, MORF4L2, PERP, and SELENBP1. The RAS family has been shown to be let-7 targets experimentally [45]. In our analysis, RASSF7 (alias HRAS1) and RAB34 (member of RAS super-family) were predicted to be let-7f targets. Specifically, RASSF7 is negatively correlated with let-7f expression among normal samples and positively correlated among tumors (Fig. 5a). The opposite pattern holds for RAB34 (Fig. 5b).

**Figure 6:**
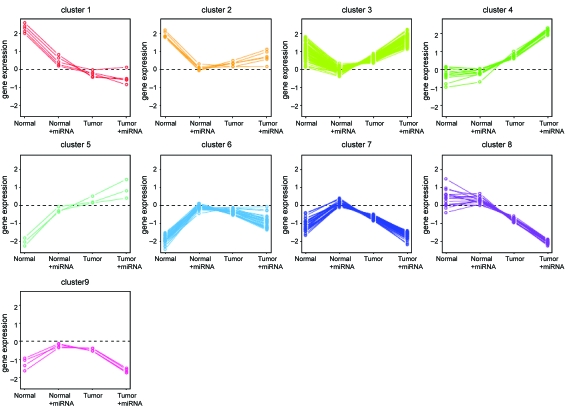
Profile plot for CORM clusters of the 178 genes predicted as let-7f targets Genes were clustered based on their relationship with let-7f and disease status using the CORM method. Each panel is for a gene cluster and each line is for a gene. X axis represents four conditions: normal, normal with one unit let-7f expression, tumor, and tumor with one unit let-7f expression. Y axis represents the average gene expression level for a given condition.

Using CORM with let-7f expression, disease status, and their interaction as covariates, the 178 genes were clustered so that genes in the same cluster vary similarly as let-7f varies given the disease status (Supplementary Table 2). Interestingly, clusters 1–4 seem to be mirror images of clusters 5–8, respectively (Fig. 6). Genes in cluster 1 are negatively correlated with let-7f in normal samples; genes in cluster 2 and 3 are negatively correlated in normal samples and positively correlated in tumors; genes in cluster 4 are positively correlated in tumors; and genes in cluster 9 are positively correlated in normal samples and negatively correlated in tumors. RASSF7 and ARAF belong to cluster 3, while RAB34 and PERP belong to cluster 6.

## Methods

### Stable hierarchical clustering

Stable hierarchical clustering groups samples based on the co-clustering frequency among repeated bootstrap sampling. Specifically, (i) bootstrap sample sets are generated by resampling with replacement from the original sample set; (ii) for each bootstrap sample set, samples were partitioned to a pre-specified number of clusters using PAM (partitioning around medoids) method and the corresponding cluster centers are applied to classify the samples in the original sample set using the nearest-centroid method; (iii) for each sample pair in the original sample set, the frequency of being assigned to the same cluster is calculated across bootstrap sample sets. The co-clustering frequency is then used as a similarity measure for hierarchical clustering to identify stable clusters of samples.

### Clustering of regression models method

As for per-gene regression analysis, CORM uses regression to model systematic variation in expression levels but, in addition, assumes that genes in the same cluster share the same values of regression coefficients [34]. To identify gene clusters, CORM was applied using the same regression model as for their per-gene analysis.

Let **X**_gi_ (n_gi_ × p) denote the design matrix for gene g and sample i, **Fβ**_k,_**ξ**_k_ the conditional distribution of genes in cluster k given the covariates with parameters **β**_k_ and **ξ**_k_, **β**_k_ (p × 1) the vector of regression coefficients, and μ(.; .) the regression function. The model underlying CORM can be written as

$$\begin{array}{c} y_{\text{gi}}|(X_{\text{gi}}{,\text{u}}_{\text{g}}=\;\text{k})∼F_{\beta \text{k},\xi \text{k}}\\ \text{E}(y_{\text{gi}}|X_{\text{gi}},{\text{u}}_{\text{g}}=\text{k})=\mu (X_{\text{gi}};\beta_{\text{k}}) \end{array}$$

where u_g_ is a random variable on (1, 2, …, K) with probabilities (π_1_, π_2_, …, π_K_). Complete specification of the CORM modeling framework requires identification of the error structure (parameterized by **ξ**), which depends on the form of the regression model. The specific form of the regression model used for CORM is flexible. For example, it can be the linear model, the linear mixed model, the nonlinear model, and the nonparametric regression model. Its choice should depend on the experimental design and the scientific question.

The clustering of linear models (CLM) method can be applied to cross-sectional data to find genes whose expression levels are similarly related to a set of covariates. In cross-sectional data, a single expression value is measured for a gene on a sample; hence, **y**_gi_ reduces to y_gi_ and **X**_gi_ to **x**_gi_. The underlying model for CLM can be written as

$$\begin{array}{c} {\text{y}}_{\text{gi}}|(x_{\text{i}},{\text{u}}_{\text{g}}=\text{k})=x^{\text{T}}{}_{\text{i}}\beta_{\text{k}}+{ɛ}_{\text{gi}}\\ {ɛ}_{\text{gi}}∼\text{N}(\text{0},\;\sigma^{\text{2}}{}_{\text{k}}) \end{array}$$

The EM algorithm can be used to fit the CLM model, and implementation details can be found in [34].

For the analysis of the human cancer data in this paper, the CLM method was used to (1) identify miRNAs similarly related to disease status and tissue type with disease status and tissue type as covariates, and (2) identify genes similarly related to let-7f and disease status with let-7f expression level, disease status, and their interaction as covariates.

CLM is closely related to K-means clustering, both being partitional clustering; however, K-means clusters gene based on the expression levels directly, while CLM based on the relationship between expression and the covariates and hence pools information across samples. Comparing to K-means, CLM tends to identify more stable clusters across samples [35].

## Discussion

The study of miRNAs has received a lot of attention lately [1, 2]. There is evidence that miRNAs are involved in animal development and cell cycle regulation. MiRNAs might play an important role in cancer and in regulating cancer-related genes [10, 11, 23, 45]. Our findings in this paper suggest the significance of microRNA expression itself in cancer diagnosis. Using let-7f as an example, we showed that its expression is correlated with the expression of a number of known oncogenes and the directionality of the correlation (positive correlation *versus* negative correlation) may be different in tumors and in normal tissues.

Target prediction is an important component in understanding miRNAs and their functions. As an alternative to existing sequence-based algorithms, an expression-based strategy for miRNA target prediction was proposed in this paper and its feasibility was demonstrated through an application to a human cancer data set. Like the sequence-based predictions, the expression-based predictions also have limitations. For example, correlation is not direct interaction and genes correlated with a miRNA might be down-stream genes of miRNA direct targets. Rather the two predictions are complementary and could be combined to prioritize candidate targets for experimental validation.

Many types of high-throughput ‘-omics’ data have recently emerged, such as gene copy number, gene expression, and proteomics data. The interpretation and integration of these data pose a challenge for both experimental and quantitative scientists in this field. The analytic methods in this paper provide a new tool to interrogate these high-throughput data in an integrative fashion. In particular, CORM has been previously applied to data collected under various experimental designs, such as cross sectional, longitudinal with no replication, and longitudinal with replications. In this paper, we demonstrated yet another application of CORM to clustering miRNAs or genes with respect to specific covariates of interests. We have focused on the cluster analysis in this paper, including the clustering of samples, miRNAs, and genes. These exploratory analyses are one aspect of an integrative analysis of miRNA and gene expression. We will investigate other types of integrative analysis in the future to gain a better understanding of the relationship between miRNAs and genes as well as their joint behaviors.

## Supplementary Material

Table S1 Sample list List of samples used in this paper.

Table S2 Predicted target genes for let-7f based on expression profiles List of the 178 genes predicted as targets for let-7f, together with their CORM cluster memberships.

## Figures and Tables

**Table 1:** The 38 cancer-related miRNAs with p-value < 0.001 and fold-change >3.

Cluster	Name	Fold-change	P-value
1	hsa-miR-23b	3.11	3.37E-09
1	hsa-miR-26b_(sub_1)	3.34	3.53E-10
1	hsa-miR-125b	3.21	7.13E-06
2	hsa-let-7f	3.27	1.40E-12
2	hsa-let-7g	3.45	4.00E-15
2	hsa-miR-15a	3.65	4.82E-10
2	hsa-miR-30c	3.65	2.96E-11
2	hsa-miR-126	4.12	5.70E-10
3	hsa-let-7d	3.79	2.89E-08
3	hsa-let-7i	3.51	3.52E-09
3	hsa-miR-19a	4.33	7.72E-10
3	hsa-miR-20_(sub_1)	3.68	6.63E-11
3	hsa-miR-28	3.75	5.73E-10
3	hsa-miR-34a_(sub_1)	3.36	3.58E-07
3	hsa-miR-101	6.37	1.41E-15
3	hsa-miR-130a	6.58	3.89E-15
4	hsa-miR-199a*	4.52	1.79E-08
4	hsa-miR-199a_(sub_1)	4.03	1.51E-05
4	mmu-miR-199b	3.68	2.62E-05
4	hsa-miR-200a	3.02	1.62E-05
4	hsa-miR-214	4.05	3.81E-07
5	hsa-miR-99a	6.99	2.78E-07
5	hsa-miR-100	5.26	3.80E-07
6	hsa-miR-33	5.56	4.82E-07
6	hsa-miR-141	4.46	3.58E-06
