# Supplementary material for: An Integrative Analysis of microRNA and mRNA Expression—A Case Study
Source: Cancer Inform. 2008 Jun 17;6:369–79. doi: 10.4137/cin.s633 (PMC2623315; doi:10.4137/cin.s633)
Supplement: Table S1 — List of samples used in this paper. [file cin-6-0369-s1.doc]

### Supplementary Table 1 – Sample list

List of samples used in this paper.

| id | sample.name | disease.status | tissue.type |
| --- | --- | --- | --- |
| 1 | N_COLON_1 | N | COLON |
| 2 | N_COLON_3 | N | COLON |
| 3 | N_COLON_4 | N | COLON |
| 4 | N_COLON_5 | N | COLON |
| 5 | T_COLON_2 | T | COLON |
| 6 | T_COLON_3 | T | COLON |
| 7 | T_COLON_4 | T | COLON |
| 8 | T_COLON_5 | T | COLON |
| 9 | T_COLON_7 | T | COLON |
| 10 | T_COLON_9 | T | COLON |
| 11 | T_COLON_10 | T | COLON |
| 12 | N_KID_1 | N | KID |
| 13 | N_KID_2 | N | KID |
| 14 | N_KID_3 | N | KID |
| 15 | T_KID_1 | T | KID |
| 16 | T_KID_3 | T | KID |
| 17 | T_KID_4 | T | KID |
| 18 | T_KID_5 | T | KID |
| 19 | N_PROST_1 | N | PROST |
| 20 | N_PROST_2 | N | PROST |
| 21 | N_PROST_4 | N | PROST |
| 22 | N_PROST_6 | N | PROST |
| 23 | N_PROST_7 | N | PROST |
| 24 | N_PROST_8 | N | PROST |
| 25 | T_PROST_1 | T | PROST |
| 26 | T_PROST_2 | T | PROST |
| 27 | T_PROST_3 | T | PROST |
| 28 | T_PROST_4 | T | PROST |
| 29 | T_PROST_5 | T | PROST |
| 30 | T_PROST_6 | T | PROST |
| 31 | N_LUNG_1 | N | LUNG |
| 32 | N_LUNG_3 | N | LUNG |
| 33 | T_LUNG_1 | T | LUNG |
| 34 | T_LUNG_2 | T | LUNG |
| 35 | T_LUNG_3 | T | LUNG |
| 36 | T_LUNG_4 | T | LUNG |
| 37 | T_LUNG_6 | T | LUNG |
| 38 | N_BRST_1 | N | BRST |
| 39 | N_BRST_2 | N | BRST |
| 40 | N_BRST_3 | N | BRST |
| 41 | T_BRST_1 | T | BRST |
| 42 | T_BRST_2 | T | BRST |
| 43 | T_BRST_3 | T | BRST |
| 44 | T_BRST_4 | T | BRST |
| 45 | T_BRST_5 | T | BRST |
| 46 | T_BRST_6 | T | BRST |
