# Supplementary material for: An Integrative Analysis of microRNA and mRNA Expression—A Case Study
Source: Cancer Inform. 2008 Jun 17;6:369–79. doi: 10.4137/cin.s633 (PMC2623315; doi:10.4137/cin.s633)
Supplement: Table S2 — List of the 178 genes predicted as targets for let-7f, together with their CORM cluster memberships. [file cin-6-0369-s2.doc]

### Supplementary Table 2 – Predicted target genes for let-7f based on expression profiles

List of the 178 genes predicted as targets for let-7f, together with their CORM cluster memberships.

| Cluster | Gene.Symbol | Gene.Title |
| --- | --- | --- |
| 1 | SELENBP1 | selenium binding protein 1 |
| 1 | MGC61598 | similar to ankyrin-repeat protein Nrarp |
| 1 | CFL1 | cofilin 1 (non-muscle) |
| 1 | ARPP-19 | Cyclic AMP phosphoprotein 19 kD |
| 1 | ALKBH7 | AlkB alkylation repair homolog 7 (E. coli) |
|  |  |  |
| 2 | PRKACG | protein kinase cAMP-dependent catalytic gamma |
| 2 | FLJ35348 | FLJ35348 |
| 2 | BCL2L14 | BCL2-like 14 (apoptosis facilitator) |
| 2 | PTK6 | PTK6 protein tyrosine kinase 6 |
| 2 | AMELY | amelogenin (amelogenesis imperfecta 1 X-linked) |
| 2 | FCBBF3021807 | CDNA FLJ34214 fis |
|  |  |  |
| 3 | GPR3 | G protein-coupled receptor 3 |
| 3 | MTMR11 | myotubularin related protein 11 |
| 3 | SMG5 | Smg-5 homolog nonsense mediated mRNA decay factor (C. elegans) |
| 3 | PIGR | polymeric immunoglobulin receptor |
| 3 | SPTBN1 | spectrin beta non-erythrocytic 1 |
| 3 | C2orf24 | chromosome 2 open reading frame 24 |
| 3 | PHF7 | PHD finger protein 7 |
| 3 | CCR9 | chemokine (C-C motif) receptor 9 |
| 3 | GAP43 | growth associated protein 43 |
| 3 | SLCO2A1 | solute carrier organic anion transporter family member 2A1 |
| 3 | MASP1 | mannan-binding lectin serine peptidase 1 (C4/C2 activating component of Ra-reactive factor) |
| 3 | GYPA | glycophorin A (MNS blood group) |
| 3 | ZNF622 | Zinc finger protein 622 |
| 3 | PCDH1 | protocadherin 1 (cadherin-like 1) |
| 3 | GABRA1 | gamma-aminobutyric acid (GABA) A receptor alpha 1 |
| 3 | DRD1 | dopamine receptor D1 |
| 3 | CREBL1 | cAMP responsive element binding protein-like 1 |
| 3 | CREBL1 /// TNXB | cAMP responsive element binding protein-like 1 /// tenascin XB |
| 3 | HLA-DOA | major histocompatibility complex class II DO alpha |
| 3 | HLA-DOB | major histocompatibility complex class II DO beta |
| 3 | BTN2A2 | butyrophilin subfamily 2 member A2 |
| 3 | URG4 | up-regulated gene 4 |
| 3 | ACHE | acetylcholinesterase (Yt blood group) |
| 3 | PMS2L1 /// PMS2L5 /// LOC441259 /// PMS2L11 /// LOC641799 /// LOC641800 /// LOC645243 /// LOC645248 | postmeiotic segregation increased 2-like 1 /// postmeiotic segregation increased 2-like 5 /// similar to postmeiotic segregation increased 2-like 2 /// postmeiotic segregation increased 2-like 11 /// similar to postmeiotic segregation increased 2-like 2 /// similar to postmeiotic segregation increased 2-like 2 /// similar to postmeiotic segregation increased 2-like 2 /// similar to postmeiotic segregation increased 2-like 2 |
| 3 | TRBV21-1 | T cell receptor beta variable 21-1 |
| 3 | NEFL | neurofilament light polypeptide 68kDa |
| 3 | POP1 | processing of precursor 1 ribonuclease P/MRP subunit (S. cerevisiae) |
| 3 | ODF1 | outer dense fiber of sperm tails 1 |
| 3 | C9orf21 | Chromosome 9 open reading frame 21 |
| 3 | PSD | pleckstrin and Sec7 domain containing |
| 3 | TTC17 | tetratricopeptide repeat domain 17 |
| 3 | PAX6 | paired box gene 6 (aniridia keratitis) |
| 3 | RASSF7 | Ras association (RalGDS/AF-6) domain family 7 |
| 3 | TUB | tubby homolog (mouse) |
| 3 | KLRK1 | killer cell lectin-like receptor subfamily K member 1 |
| 3 | ACVR1B | activin A receptor type IB |
| 3 | SILV | silver homolog (mouse) |
| 3 | SMARCD1 | SWI/SNF related matrix associated actin dependent regulator of chromatin subfamily d member 1 |
| 3 | GSTZ1 | glutathione transferase zeta 1 (maleylacetoacetate isomerase) |
| 3 | TRAF3 | TNF receptor-associated factor 3 |
| 3 | ITGAD | integrin alpha D |
| 3 | SPN | sialophorin (leukosialin CD43) |
| 3 | SCNN1G | sodium channel nonvoltage-gated 1 gamma |
| 3 | C16orf45 | Chromosome 16 open reading frame 45 |
| 3 | AYTL1 | acyltransferase like 1 |
| 3 | GNAO1 | guanine nucleotide binding protein (G protein) alpha activating activity polypeptide O |
| 3 | CALB2 | calbindin 2 29kDa (calretinin) |
| 3 | COX10 | COX10 homolog cytochrome c oxidase assembly protein heme A: farnesyltransferase (yeast) |
| 3 | P2RX5 | purinergic receptor P2X ligand-gated ion channel 5 |
| 3 | MGC4172 | short-chain dehydrogenase/reductase |
| 3 | GFAP | glial fibrillary acidic protein |
| 3 | ATP6V0A1 | ATPase H+ transporting lysosomal V0 subunit a1 |
| 3 | ITGA3 | integrin alpha 3 (antigen CD49C alpha 3 subunit of VLA-3 receptor) |
| 3 | PNMT | phenylethanolamine N-methyltransferase |
| 3 | CHERP | calcium homeostasis endoplasmic reticulum protein |
| 3 | ATP4A | ATPase H+/K+ exchanging alpha polypeptide |
| 3 | ATP1A3 | ATPase Na+/K+ transporting alpha 3 polypeptide |
| 3 | IGL@ | Immunoglobulin lambda locus |
| 3 | PPM1F | protein phosphatase 1F (PP2C domain containing) |
| 3 | DUSP18 | dual specificity phosphatase 18 |
| 3 | TXN2 | thioredoxin 2 |
| 3 | SSTR3 | somatostatin receptor 3 |
| 3 | SSX2 /// LOC653088 | synovial sarcoma X breakpoint 2 /// similar to synovial sarcoma X breakpoint 2 isoform b |
| 3 | ARAF | v-raf murine sarcoma 3611 viral oncogene homolog |
| 3 | APLN | apelin AGTRL1 ligand |
| 3 | ZWINTAS | ZW10 interactor antisense |
| 3 | --- | PTR2 mRNA for repetitive sequence |
| 3 | --- | MRNA full length insert cDNA clone EUROIMAGE 146729 |
| 3 | --- | --- |
| 3 | --- | --- |
| 3 | --- | --- |
| 3 | --- | --- |
| 3 | --- | --- |
| 3 | --- | --- |
| 3 | --- | --- |
|  |  |  |
| 4 | GPATC3 | G patch domain containing 3 |
| 4 | CD34 | CD34 molecule |
| 4 | INPP5E | inositol polyphosphate-5-phosphatase 72 kDa |
| 4 | GLCE | UDP-glucuronic acid epimerase |
| 4 | STARD3 | START domain containing 3 |
| 4 | CNP | 2' 3'-cyclic nucleotide 3' phosphodiesterase |
| 4 | MAP4K1 | mitogen-activated protein kinase kinase kinase kinase 1 |
| 4 | C20orf4 | chromosome 20 open reading frame 4 |
| 4 | ZNF317 | zinc finger protein 317 |
| 4 | --- | Transcribed locus moderately similar to XP_517655.1 PREDICTED: similar to KIAA0825 protein [Pan troglodytes] |
| 4 | --- | --- |
|  |  |  |
| 5 | EHBP1 | EH domain binding protein 1 |
| 5 | ZC3HAV1 | zinc finger CCCH-type antiviral 1 |
| 5 | SERPINH1 | serpin peptidase inhibitor clade H (heat shock protein 47) member 1 (collagen binding protein 1) |
|  |  |  |
| 6 | PHGDH | phosphoglycerate dehydrogenase |
| 6 | SFRS4 | splicing factor arginine/serine-rich 4 |
| 6 | CCNL2 /// LOC643556 | cyclin L2 /// similar to Aurora kinase A-interacting protein (AURKA-interacting protein) |
| 6 | SLC35E2 | solute carrier family 35 member E2 |
| 6 | CLK2 | CDC-like kinase 2 |
| 6 | TOMM20 | translocase of outer mitochondrial membrane 20 homolog (yeast) |
| 6 | ASCC3L1 | activating signal cointegrator 1 complex subunit 3-like 1 |
| 6 | C2orf12 /// LOC648293 | chromosome 2 open reading frame 12 /// region containing chromosome 2 open reading frame 12; RNA binding motif single stranded interacting protein 1 |
| 6 | LAMB2 | laminin beta 2 (laminin S) |
| 6 | SPCS1 | signal peptidase complex subunit 1 homolog (S. cerevisiae) |
| 6 | SEC22C | SEC22 vesicle trafficking protein homolog C (S. cerevisiae) |
| 6 | RPL14 | ribosomal protein L14 |
| 6 | EIF2A | eukaryotic translation initiation factor 2A 65kDa |
| 6 | CTBP1 | C-terminal binding protein 1 |
| 6 | CCNI | cyclin I |
| 6 | HIST1H2BK | histone 1 H2bk |
| 6 | C6orf111 | chromosome 6 open reading frame 111 |
| 6 | PERP | PERP TP53 apoptosis effector |
| 6 | GTF3C4 | General transcription factor IIIC polypeptide 4 90kDa |
| 6 | FER1L3 | fer-1-like 3 myoferlin (C. elegans) |
| 6 | BANF1 /// LOC645870 | barrier to autointegration factor 1 /// similar to barrier to autointegration factor 1 |
| 6 | CD276 | CD276 molecule |
| 6 | RAB34 | RAB34 member RAS oncogene family |
| 6 | MTMR4 | myotubularin related protein 4 |
| 6 | RBX1 | ring-box 1 |
| 6 | clone NT2RP2005980 | CDNA FLJ14188 fis |
| 6 | --- | --- |
| 6 | --- | --- |
| 6 | --- | --- |
| 6 | --- | --- |
|  |  |  |
| 7 | HIAT1 | hippocampus abundant transcript 1 |
| 7 | BSDC1 | BSD domain containing 1 |
| 7 | C2orf30 | chromosome 2 open reading frame 30 |
| 7 | JAGN1 | jagunal homolog 1 (Drosophila) |
| 7 | FSTL1 | follistatin-like 1 |
| 7 | RPL35A | ribosomal protein L35a |
| 7 | TBCA | tubulin-specific chaperone a |
| 7 | HSP90AB1 | heat shock protein 90kDa alpha (cytosolic) class B member 1 |
| 7 | MLL3 | myeloid/lymphoid or mixed-lineage leukemia 3 |
| 7 | STX17 | syntaxin 17 |
| 7 | NDUFC2 | NADH dehydrogenase (ubiquinone) 1 subcomplex unknown 2 14.5kDa |
| 7 | KRT5 | keratin 5 (epidermolysis bullosa simplex Dowling-Meara/Kobner/Weber-Cockayne types) |
| 7 | CDK2AP1 | CDK2-associated protein 1 |
| 7 | METTL3 | methyltransferase like 3 |
| 7 | ACTN1 | actinin alpha 1 |
| 7 | DLST /// PA2G4 | dihydrolipoamide S-succinyltransferase (E2 component of 2-oxo-glutarate complex) /// proliferation-associated 2G4 38kDa |
| 7 | RPL4 | ribosomal protein L4 |
| 7 | MGC18216 | hypothetical protein MGC18216 |
| 7 | MT1M /// MT2A /// LOC441019 | metallothionein 1M /// metallothionein 2A /// hypothetical gene supported by X97260; BC070289 |
| 7 | GLG1 | golgi apparatus protein 1 |
| 7 | SNTB2 | syntrophin beta 2 (dystrophin-associated protein A1 basic component 2) |
| 7 | LOC339287 | hypothetical protein LOC339287 |
| 7 | RPL18A | ribosomal protein L18a |
| 7 | CHERP | calcium homeostasis endoplasmic reticulum protein |
| 7 | RNF12 | Ring finger protein 12 |
| 7 | MORF4L2 | mortality factor 4 like 2 |
| 7 | REPS1 | RALBP1 associated Eps domain containing 1 |
| 7 | --- | --- |
|  |  |  |
| 8 | PRDX1 | peroxiredoxin 1 |
| 8 | SERINC2 | serine incorporator 2 |
| 8 | FLJ14668 | hypothetical protein FLJ14668 |
| 8 | FLJ20254 | hypothetical protein FLJ20254 |
| 8 | IQSEC1 | IQ motif and Sec7 domain 1 |
| 8 | HSPA1A | heat shock 70kDa protein 1A |
| 8 | C6orf111 | chromosome 6 open reading frame 111 |
| 8 | LSM5 | LSM5 homolog U6 small nuclear RNA associated (S. cerevisiae) |
| 8 | COX6C | cytochrome c oxidase subunit VIc |
| 8 | MGEA5 | meningioma expressed antigen 5 (hyaluronidase) |
| 8 | KIAA0692 | KIAA0692 |
| 8 | COPS2 | COP9 constitutive photomorphogenic homolog subunit 2 (Arabidopsis) |
| 8 | PSMA4 | proteasome (prosome macropain) subunit alpha type 4 |
| 8 | PRPSAP1 | phosphoribosyl pyrophosphate synthetase-associated protein 1 |
| 8 | TXNL4A | thioredoxin-like 4A |
| 8 | --- | --- |
|  |  |  |
| 9 | CCT5 | chaperonin containing TCP1 subunit 5 (epsilon) |
| 9 | PSMA6 | proteasome (prosome macropain) subunit alpha type 6 |
| 9 | HM13 | histocompatibility (minor) 13 |
| 9 | --- | --- |
